# A mini-review on the emerging role of nanotechnology in revolutionizing orthopedic surgery: challenges and the road ahead

**DOI:** 10.3389/fbioe.2023.1191509

**Published:** 2023-05-16

**Authors:** Yongjun Deng, Chao Zhou, Lifeng Fu, Xiaogang Huang, Zunyong Liu, Jiayi Zhao, Wenqing Liang, Haiyan Shao

**Affiliations:** ^1^ Department of Orthopedics, Affiliated Hospital of Shaoxing University, Shaoxing, China; ^2^ Department of Orthopedics, Zhoushan Guanghua Hospital, Zhoushan, China; ^3^ Department of Orthopedics, Shaoxing City Keqiao District Hospital of Traditional Chinese Medicine, Shaoxing, China; ^4^ Department of Orthopedics, Zhoushan Hospital of Traditional Chinese Medicine Affiliated to Zhejiang Chinese Medical University, Zhoushan, China

**Keywords:** nanotechnology, nanoparticle, orthopedic surgery, orthopedics, bone healing

## Abstract

An emerging application of nanotechnology in medicine currently being developed involves employing nanoparticles to deliver drugs, heat, light, or other substances to specific types of cells (such as cancer cells). As most biological molecules exist and function at the nanoscale, engineering and manipulating matter at the molecular level has many advantages in the field of medicine (nanomedicine). Although encouraging, it remains unclear how much of this will ultimately result in improved patient care. In surgical specialties, clinically relevant nanotechnology applications include the creation of surgical instruments, suture materials, imaging, targeted drug therapy, visualization methods, and wound healing techniques. Burn lesion and scar management is an essential nanotechnology application. Prevention, diagnosis, and treatment of numerous orthopedic conditions are crucial technological aspects for patients’ functional recovery. Orthopedic surgery is a specialty that deals with the diagnosis and treatment of musculoskeletal disorders. In recent years, the field of orthopedics has been revolutionized by the advent of nanotechnology. Using biomaterials comprised of nanoparticles and structures, it is possible to substantially enhance the efficacy of such interactions through nanoscale material modifications. This serves as the foundation for the majority of orthopedic nanotechnology applications. In orthopedic surgery, nanotechnology has been applied to improve surgical outcomes, enhance bone healing, and reduce complications associated with orthopedic procedures. This mini-review summarizes the present state of nanotechnology in orthopedic surgery, including its applications as well as possible future directions.

## 1 Introduction

Richard Feynman recognized the potential positive ramifications of nano concepts as early as 1959. Norio Taniguchi first used the word “nanotechnology” in 1974, and Eric Drexler’s writings helped popularize the concept (Sullivan et al., 2014a; Chen, 2022). An emerging application of nanotechnology in medicine currently being developed involves employing nanoparticles to deliver drugs, heat, light, or other substances to specific types of cells (such as cancer cells). As the majority of biological molecules exist and perform at the nanoscale, engineering and manipulating substances at the molecular level provides various benefits in medicine (nanomedicine). Although promising, it remains unclear how much of this will ultimately result in improved patient care. Concerns regarding the safety and cost-effectiveness of nanotechnology remain ambiguous (Kennedy et al., 2022). By permitting the simultaneous delivery of photoreactive agents and other agents (such as anticancer medications, etc.), NPs play a crucial role in cancer therapy. NPs can be used as photothermal therapy (PTT) and photodynamic therapy (PDT) agents due to their delayed degradation, controlled release, optimal surface functionality, and high optical absorbance (Sargazi et al., 2022a). Similarly, NPs are very promising for diagnosing, and aptamer-conjugated carbon-based nanomaterials are proven effective for cancer and bacteria theranostics (Sargazi et al., 2022b). Advances in nanomaterials also bring revolution in modulating the tumor microenvironment and diagnosis of various tumors (Arshad et al., 2022; Wang et al., 2023), and acute respiratory diseases (Zhang et al., 2022). Continued efforts are underway in nanomaterials designing and optimization for better therapeutic benefits (Lan et al., 2021; Wang et al., 2021).

Orthopaedics is an interesting area in which nanotechnology can be used because bone and its components, like Haversian systems, hydroxyapatite, and collagen fibrils are nano-compounds (Lantieri et al., 2023). In orthopedic operations, host tissue and biomaterials frequently have a micro-level relationship. Using biomaterials comprised of NPs and structures, it is possible to substantially enhance the efficacy of such interactions through nanoscale material modifications (Wang and Tang, 2019). This serves as the foundation for the majority of orthopedic nanotechnology applications. Nanotechnology’s application to orthopedic research is prospective because it allows for improving the mechanical characteristics and biocompatibility of implanted orthopedic equipment. Implants and prostheses with nanostructures offer improved mechanical strength, increased resistance to erosion and corrosion, the potential for medication delivery, and the potential to serve as scaffolds for tissue restoration (Kienapfel et al., 1999; Bishop et al., 2012; Hanc et al., 2016).

**FIGURE 1:**
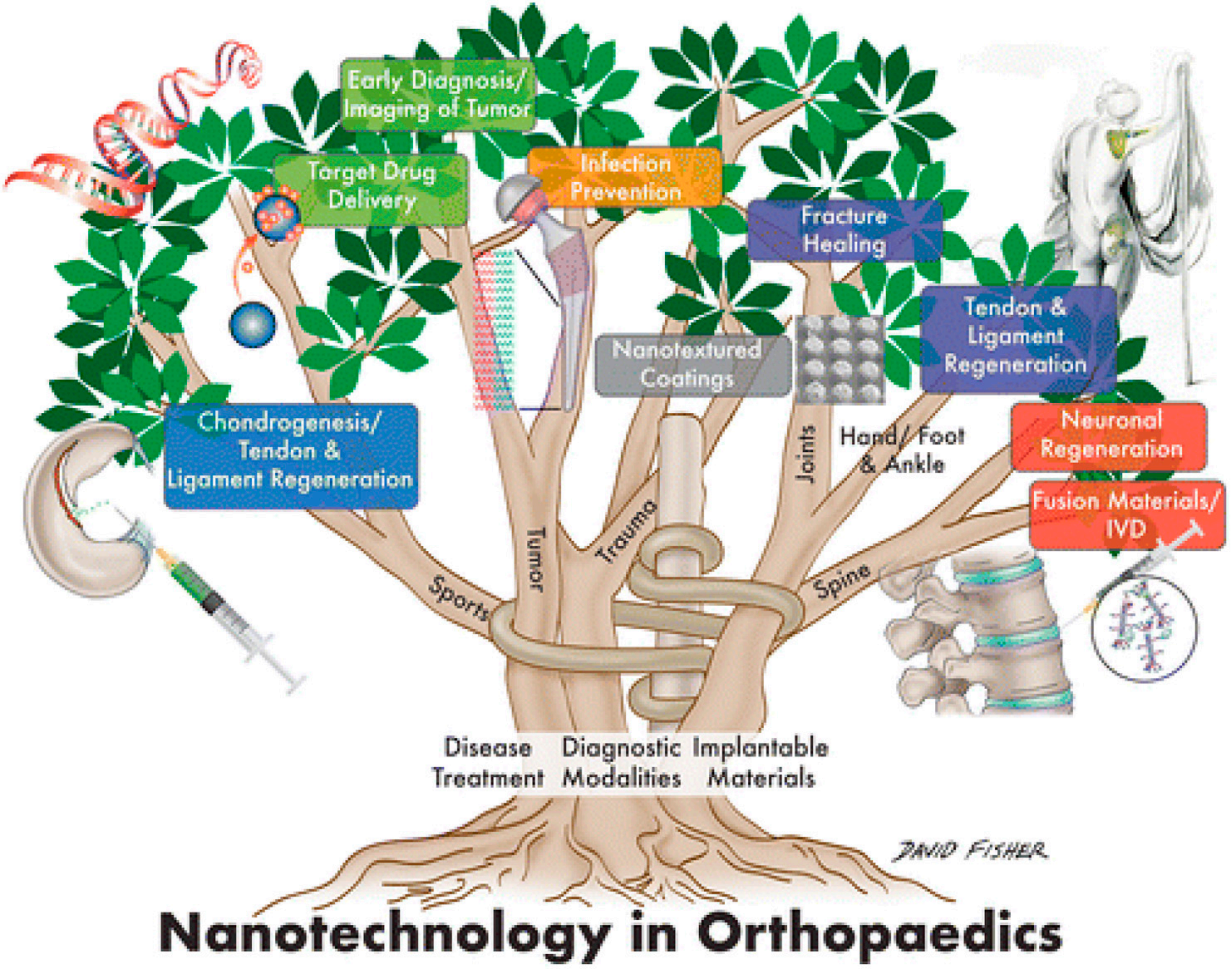
Schematic illustration showing the application of nanotechnology for the Orthopedic (Smith et al., 2018).

More efficient antibiotic and chemotherapeutic medication delivery systems, 2) Implant and prosthesis surface preparations to increase osteointegration and decrease biofilm development, 3) Regulated medication eluting systems are being developed to address infections caused by implants, 4) Tissue engineering (TE) has been employed to build scaffolds for dealing with cartilage and bone abnormalities, and 5) Oncology-related applications for diagnostics are the major orthopedic applications of nanotechnology. Figure 1 gives a schematic overview of the nanotechnology based approaches for diagnosing and treating various orthopedics issues. The application of nanotechnology to medicine, also known as “nanomedicine,” has been utilized in various novel orthopedic therapies. Several clinical applications of nanotechnology include targeted drug delivery, implantable materials, vertebral disk regeneration, and diagnostic modalities. Nanotechnology enables more precise treatment modalities, which may result in more effective and durable implants, decreased infection rates, and enhanced bone and tendon regeneration. Particularly in the field of orthopedics, the theoretical benefits of nanomedicine are beginning to be realized due to massive efforts in fundamental science research (Garimella and Eltorai, 2017a; Smith et al., 2018). Table 1 enlisted various contemporary NPs that are reported in orthopedic surgeries. This review summarizes the present state of nanotechnology in orthopedic surgery, including its applications as well as possible future directions. This will be helpful for the researcher to distill this information for future studies and update the readers and healthcare providers in the field of orthopedics.

**TABLE 1:** Examples of contemporary nanomedicine use in orthopedic surgeries.

Applications	Medical condition	Nanocomponent	References
Drug delivery	Tendonitis is an inflammation of a tendon (the tight cords of tissue that link muscles to bones), resulting in pain, discomfort, and swelling	Autologous tenocyte injection has been demonstrated to increase tendon remodeling, tensile strength, and collagen contents. The incorporation of tenocytes was monitored using nanoparamagnetic iron oxide	Chen et al. (2011)
Diagnostics	Herniated disc—occurs when an intervertebral disc starts to deteriorate and the internal nucleus bursts. The herniated disc material segments then compress on the nerve roots, producing pain, numbness, weakness, or alterations in sensation	Mechanical measurement of nanoscale characteristics of the annulus fibrosis using atomic force. The nanoscale strain/stress and hydration characteristics were investigated. A significant step forward in giving tissue substitutes in the case of spinal injury in comparison to the present mechanical prosthesis	Lewis et al. (2008)
Biomaterials	Osteoarthritis is characterized largely by cartilage breakdown and joint space narrowing; it can also involve bone overgrowth, spur development, and impaired function	Anodized TiO2 nanotube surface constructions reduced nitrous oxide generation and fibrotic capsule development	Smith et al. (2011)
		Many implants and joint restorations fail as a result of extensive fibrotic capsule development	
Therapeutics	Rheumatoid arthritis is an inflammatory condition that impacts the joint lining (synovium). The inflammation might impact all of the joints	Inner hollow nanospheres in a chemically altered chondroitin sulphate solution. The spheres will add therapeutic characteristics to standard chondroitin sulphate treatment by incorporating medication or growth methods	
Imaging	Scoliosis is characterized by sideways, or lateral, curvature and rotations of the back bones (vertebrae), creating the impression that the individual is leaning to one side	Neuro-central growth plate nanostructure imaging. Problems could be found as small as a nanometer and are known to induce idiopathic scoliosis as well as other neurocentral growth plate illnesses	Hauge Bünger et al. (2006)
Biomaterials	Osteoporosis is characterized by bone mass loss and bone tissue breakdown. This process weakens the bones and makes them more prone to breaking	A bone morphogenetic protein-loaded nano-HA ceramic/polymer composite (BMP-7) provides long-term medication delivery and bone scaffolding	Liu and Webster (2010)
Implants	Osteomyelitis (postoperative)—infection triggered by contamination of the injured area, frequently resulting from bacterial adhesion to implants	Sharklet AFTM, a surface microtopography based on shark skin, has been proven to disrupt the production of bacterial biofilms without applying bacteriocidal chemicals	Chung et al. (2007)
Biomaterials	Fracture—a partial or complete fracture or break through a bone caused by impact damage	Healing of a radial lesion with a nano-HA bone scaffold with holes ranging from 100 to 250 lm. The porous nano-HA improved bone growth and biocompatibility	Zhu et al. (2009)
Drug delivery	Osteosarcoma is an aggressive malignancy that often manifests in children and teens	In osteosarcoma patients, a polymeric nanosystem is used to administer doxorubicin	Susa et al. (2009)
		Its therapeutic effect is dependent on enhanced doxorubicin accumulation in the nucleus. This approach has shown potential in treating multidrug-resistant osteosarcoma cell lines	
Sensor	Fracture—a partial or complete fracture or break in a bone caused by an impact damage	Multiwalled carbon nanotubes will be capable of detecting new bone development *in vivo*. As the electrical characteristics of bone vary from those of surrounding tissue as well as fibrous scars, it is feasible to examine the electrical conductivity of new development to determine which tissues are forming. This data can then be transmitted to a radio frequency receiver located external to the body	Brenner and Ling (2012)

## 2 Nanotechnology in prosthetic joint replacement

Osteointegration failure is one of the primary concerns associated with the increased usage of uncemented complete arthroplasties of the joints. Even though joints of prosthetics are now treated for surface irregularity to enhance osseous ongrowth or ingrowth, the nanoscale, where cellular interactions occur, remains smooth. This promotes fibrous instead of bony ingrowth, resulting in premature failure (Katz et al., 2013). Utilizing nanoengineered implants and nanotextured surfaces will aid in resolving the issue by enhancing osteoblastic cell activity. The enhanced surface area of nanoengineered implants enabled improved connections between the surface of the implant and the host bone, opening the path for reliable and predicted osteointegration and extending the implants’ lifespan.

One of the leading reasons for early joint replacement failure and revision is periprosthetic joint infection. Several approaches, including antibiotic-loaded cement as well as other localized drug delivery systems, have been utilized with varying degrees of achievement. It has been demonstrated that the employment of titanium (Ti) nanotubes for nanophase silver or polypeptide nanofilm coatings on prosthetic surfaces regulated and sustained antibiotic release after the operation is efficient. It has been demonstrated that bacterial adhesion and colonization are diminished. Therefore, regulated antibiotic-eluting nanophasic prosthetic joints are a possible remedy for the catastrophic threat posed by periprosthetic joint infection (Gusić et al., 2014a).

## 3 Drug delivery system using nanotechnology

Nanotechnology has revolutionized therapeutics by enabling more precise drug delivery, which has proven notably advantageous in orthopedics. As mentioned previously, this is accomplished in part by combining medication delivery with nanosensors. Nanophase delivery methods, on the other hand, can be employed to deliver drugs without a sensor.

**FIGURE 2:**
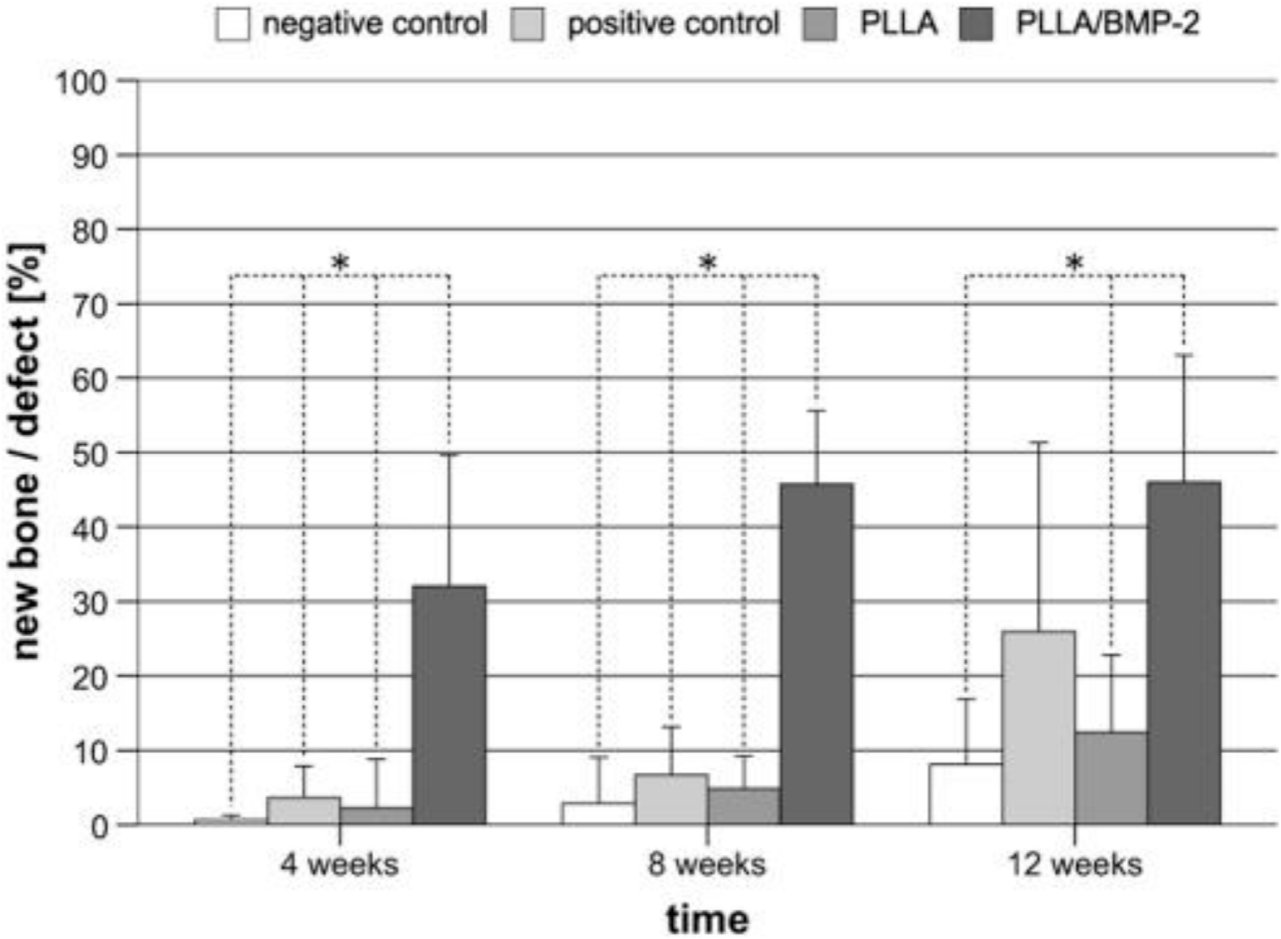
Histomorphometry shows the formation of new bone following the implantation of PLLA and control groups over a time period (Schofer et al., 2011).

Using nanotechnology and gold NPs, precise drug delivery has shown extremely promising outcomes. Animal research studies have demonstrated that gold has the potential to effectively deliver iontophoresis and is used to cure tendinopathy or a tendon illness and injury (Dohnert et al., 2012; Sullivan et al., 2014b). It has also been demonstrated that nanophase gold improves the efficacy of certain anti-inflammatory agents in rodents (Dohnert et al., 2012). Other nanoscale drug delivery methods, such as poly-l-lactic acid (PLLA), have demonstrated promise. PLLA has been employed to deliver bone morphogenetic protein (BMP), thereby accelerating the closure of certain significant bone deformities (Schofer et al., 2011). A previous study showed that PLLA implantation results in rapid filling of the bone and a hard callus was observed after 4 weeks (Figure 2) (Schofer et al., 2011).

For possible TJR uses, nanophase medication delivery is being examined. In a study conducted by Li et al., a nanofilm composed of a biodegradable polypeptide was employed to deliver cefazolin; the findings of this research investigation suggest that nanoscale medication delivery for TJR might help lower the infection risk while also increasing osteoblast recruitment and bone formation. (Li et al., 2010; Sullivan et al., 2014b). This system is of particular interest because it might also be able to modify the pharmacokinetics of antibiotic administration and time their release to occur during the most crucial phase, shortly after implantation. (Li et al., 2010).

Nanotechnology-based drug delivery is also applicable to tumor treatment, especially cancer of the bones. Osteosarcoma is a prevalent type of tumor, and the bone is a major site of cancer metastasis (Kansara and Thomas, 2007). Current investigation has uncovered nanomaterial that can specifically recognize and deliver chemotherapy to malignant bones. (Mohamed et al., 2012). A collagen hydroxyapatite biocomposite enhanced with magnetite for bone transplantation, a 3D nanomagnetite-chitosan rod for local cryotherapy, and a magnetite-hydroxyapatite composite for direct bone bonding are among the latest monotherapies (Hu et al., 2006; Murakami et al., 2008; Andronescu et al., 2010).

Recently, nanotechnology for targeted drug therapy has incorporated tiny particles (such as silver) into materials with nanostructures (Wei et al., 2006; Xing et al., 2011; Pleshko et al., 2012). Using nanotechnology for drug delivery enhances accuracy and reduces bacterial proliferation, thereby decreasing the risk of infection.

## 4 Orthopedic oncology and nanotechnology

Nanotechnology’s orthopedic oncological applications have significant potential to enhance diagnosis, overcome medication resistance, decrease the toxicity of normal host cells, as well as more efficiently deliver medications to tumor cells (Savvidou et al., 2016). NPs are capable of transporting ligands. The addition of particular ligands that bond to the unique genes expressed by tumor cells might improve the capability to detect primary as well as malignant metastasis bone tumors promptly and accurately. Contrast agent-loaded NPs can boost the precision of specific targeting cancer imaging and the assessment of cancer viability, which can be quite beneficial for preoperative evaluation and surgery planning. Multidrug resistance proteins (MDR) on the surface of cancer cells provide resistance, which transports cancer drugs out of the cells and diminishes their intracellular levels. Vehicles can be developed using nanotechnology that efficiently transports anti-cancer pharmaceuticals into the cell, while simultaneously delivering particular sequences of genes to combat MDR proteins (Barani et al., 2021; Liao et al., 2021). Nanophase drug delivery systems enhance both passive and active tumor cell targeting. Following endocytosis, drug-loaded NPs can be combined with surface ligands such as folic acid and mannose (active targeting) to determine the specific target tumor cell. Under their diminutive size (passive targeting) and by exploiting the permeability of tumor cells, NPs also permit higher drug concentrations within cancer cells. By downregulating specific genes, nanotechnology can also enhance our capacity to prevent the onset of cancer. Certain fusion oncogenes and molecular markers linked with Ewing’s sarcoma and osteosarcoma can be dysregulated utilizing nanostructures packed with particles targeted to suppress these molecular indicators (Liao et al., 2021).

**FIGURE 3:**
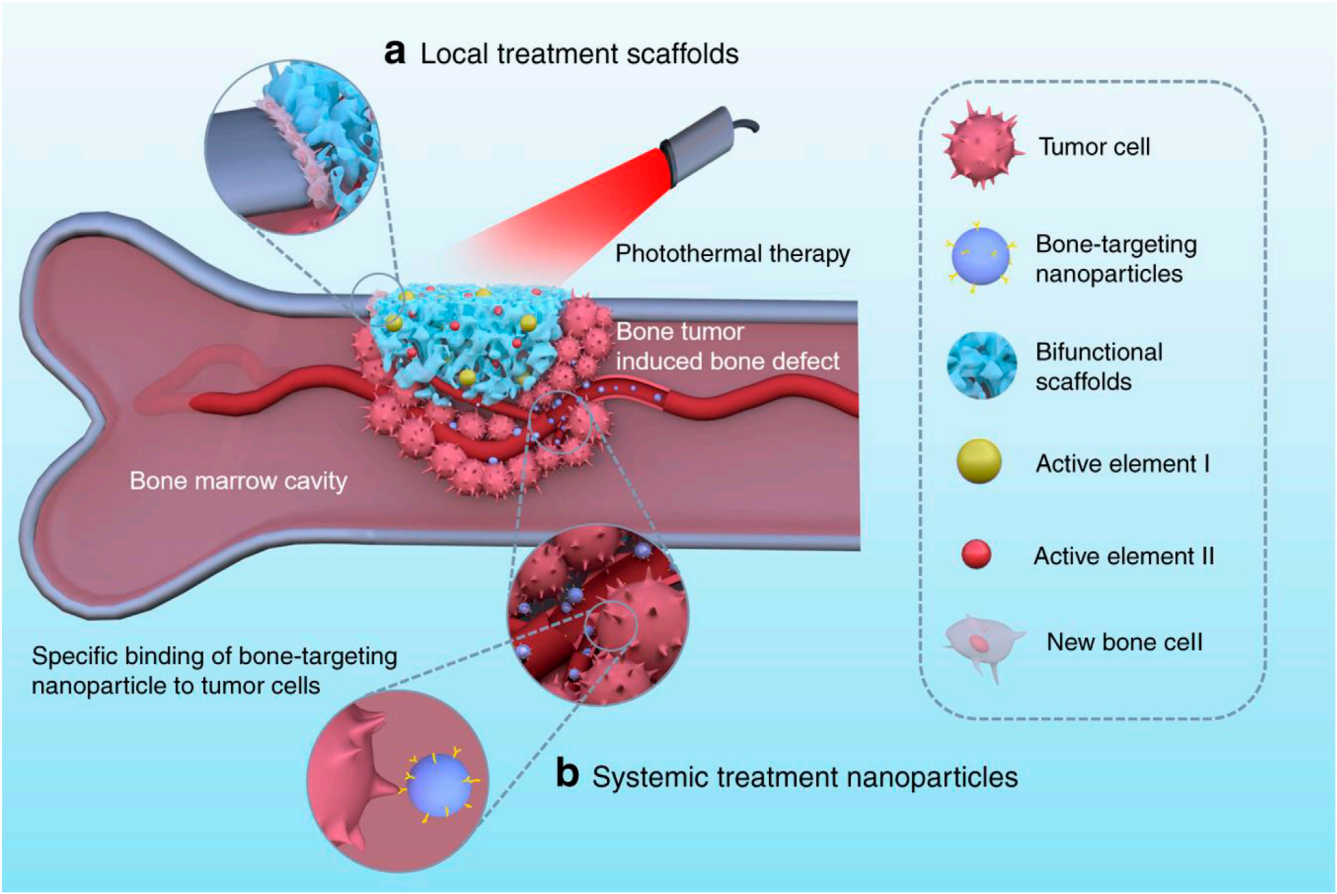
For tumor photothermal therapy and bone regeneration, bifunctional biomaterials comprise **(A)** local treatment scaffolds such as 3D-printed scaffolds, nano/microparticle-containing scaffolds, and hydrogels, and **(B)** systemic treatment nanoparticles like bone-targeting nanoparticles (Liao et al., 2021).

The bone tumor can be eradicated using either local or systemic bifunctional biomaterials. The locally applied bifunctional scaffolds, which included hydrogels, nano/microparticle-containing scaffolds, and 3D-printed scaffolds, can be placed into the area of the bone defect for tumor photothermal treatment, promoting bone healing. When delivered systemically, nanoparticles target bone tissues for tumour therapy and prevent bone reabsorption by penetrating blood arteries (Figure 3).

## 5 Nanotechnology in the cure of chondral and osseous deformities

Therapy of segmental bone deformities caused by arthroplasty, and trauma-failed fixations presents a formidable obstacle. Present methods for treating these abnormalities with porous metals and auto/allografts have constraints, like restricted availability, risk of infection, and insufficient scaffolding abilities, which limits the degree of osteointegration. While the best scaffold for promoting osteointegration is controlled by the level of contact between the host tissues and the biomaterial, nanomaterials biomaterials are ideal because osteoblasts can colonize them (Andreacchio et al., 2018). On the ultimate scaffolds, cells can interrelate, propagate, and metamorphose into natural tissues.

Biomaterials with nanostructures can offer structural support as well as appropriate pore size, as well as serve as a substrate for cell activity and movement. They can also send biological signals to control tissue transformation when laden with chemokines and growth factors, along with pharmacological assistance by providing peptide patterns that bind receptors and activate intracellular signaling cascades. Nanomaterials with these properties are regarded optimal for treating large bone deformities (Luthringer et al., 2013; Roddy et al., 2018). After their structural, biochemical, biological, and templating processes are completed, Nanoscaffolds will degrade over time, enabling a more natural restoration without the difficulties related to implants and non-disintegrating biomaterials (Roddy et al., 2018).

Many naturally occurring and manmade nanostructured substances have been explored to cure bone deformities. Although natural biomaterials offer good biocompatibility, their handling properties, and structural support are substandard. In contrast, synthetic materials provide exceptional structural assistance but are not biocompatible. Synthetic biomaterials such as bioactive ceramics (hydroxyapatite (HA) and tricalcium phosphate (TCP) and derivatives), polymers including poly-glycolic acid (PGA) and poly-lactic acid (PLA), and a mixture of these known as composite matrices are currently favored as scaffolding substances for curing bone abnormalities because of their improved structural assistance. Surface therapy of these nanostructured biomaterials with growth factors, including bone sialoproteins (BSP) and bone morphogenic proteins (BMP), can enhance their capacity for effective osteointegration. Natural polymers such as gelatin and fibrin have also been utilized to treat bone deformities in non-load-bearing areas, for instance, cranial defects.

Cartilage has a more complex structure, making it more difficult to treat cartilaginous defects with biological or synthesized scaffolds. Because of their greater biocompatibility, biodegradability, neovascularization, and cell infiltration (Vasita and Katti, 2006), biological protein scaffolds like polysaccharide and collagen scaffolds, including chitosan, hyaluronic acid, chondroitin sulphate, and agarose, are favored for the treatment of cartilage problems. Despite their immunoreactivity, type I collagen scaffolds are the most common. Acid-treated collagen polymers comprising MSCs have been demonstrated to create hyaline-like cartilage in individuals with chondral abnormalities. Gelatin is a denatured replacement for collagen that is immune-reactive and disease-transmissible.

Although most cartilage lesions may be repaired with less invasive surgery, the availability of parenteral scaffolds is crucial. Hydrogels are injectable nanomaterial polymeric networks made of collagen or gelatin that may solidify and acquire the appropriate defect form after insertion. When injected with chondrocytes, hydrogels have been demonstrated to form a cartilage-like extracellular matrix (ECM) with increasing technological advancement characteristics due to the continual accumulation of glycosaminoglycan-rich matrix.

Using nanofibers to create chondrogenic or osteogenic scaffolds has demonstrated numerous benefits, including enhanced cell adhesion, propagation, and migration. The greatest concentration of type II collagen was seen in nanofiber scaffolds and improved absorption of human serum proteins, as well as considerable activation of cartilage-specific genes and proteins, including collagen II and IX. Numerous published investigations have demonstrated that tissue engineering for treating cartilage and osseous abnormalities is one of the most significant uses of nanotechnology and associated investigation in orthopedics (D’Antimo et al., 2017).

## 6 Arthroplasty

### 6.1 Implant material

**FIGURE 4:**
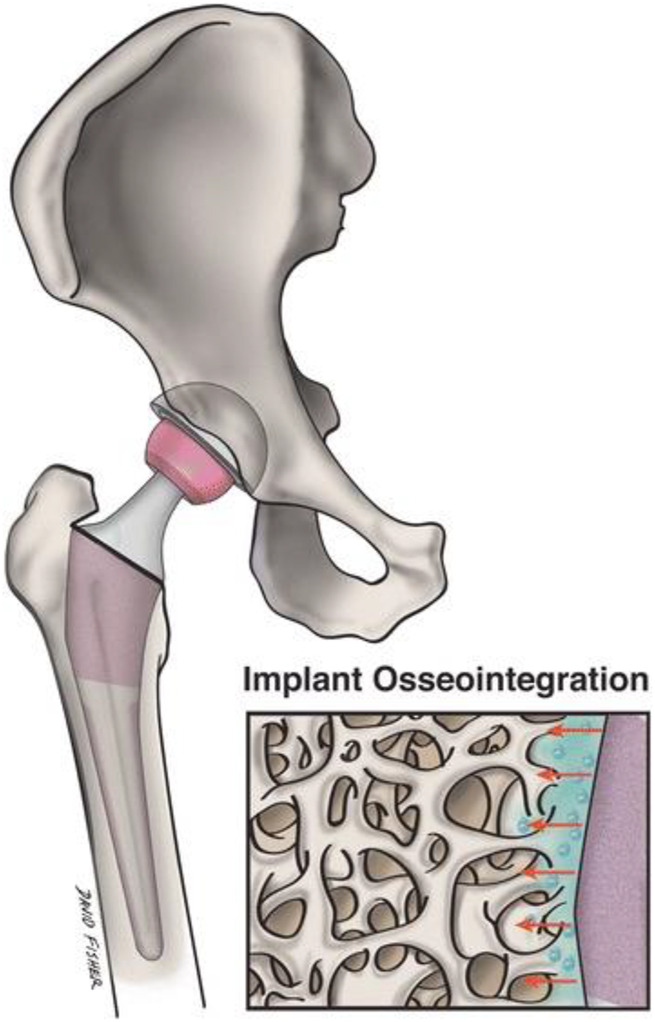
Nanostructured implants might effectively replicate the native bone surroundings and boost implant osseointegration and adjacent osteogenesis more than traditional implants. This image depicts a magnified nanoengineered implant surface and its topographical connection with the surrounding bone.

Although primary replacement surgery for joints has a high success rate, its durability is restricted. In arthroplasty, nanotechnology focuses on creating implanted materials able to function safely and efficiently while extending the typical lifetime of implants and preventing infection. By altering specific surface characteristics of the graft, a more beneficial interaction between the implant and native bone can be induced (Figure 4). Nanotextured implant surfaces have increased implant osseointegration by enhancing osteoblast function and growth (Gusić et al., 2014b). The technique of severe plastic deformation (SPD) in particular has shown the capability to enhance Ti implants’ mechanical and biocompatibility qualities (Serra et al., 2013). SPD decreases metal coarse granules to the nanoscale range by subjecting the metal to a complicated high-stress condition. In the field of arthroplasty, the usage of ultra-high molecular weight polyethylene (UHMWPE) grafts has been restricted out of concern for potential fracture. Because of UHMWPE’s biocompatibility and wear resistance, there has been considerable interest in improving its mechanical strength using nanotechnology. Carbon nanotubes were incorporated into this material to generate a unique composite, which may 1 day be helpful as an acetabular liner or tibial component (Puértolas and Kurtz, 2014). Modifying the nanostructure of an implant’s surface can boost resistance to static and dynamic fatigue, enhance functioning, and extend implant survival.

## 7 Diagnosis

Another important application of nanotechnology in orthopedics is detection. In particular, nanotechnology has been applied to diagnose bone ailments like renal osteodystrophy, osteoporosis, and Paget’s disease (Yun et al., 2009). This is frequently achieved using biosensors. These implantable sensors can be found in numerous designs and configurations. Carbon nanotubes (CNTs) are frequently used in biosensors because the sensor’s distinct characteristics render them both resilient and electrically conductive (Lin et al., 2004).

Numerous nanotechnology-based detection products are revolutionizing the orthopedics industry. In the case of osteoporosis, for instance, diagnostic techniques must provide accurate data detection in a fast, cost-effective, and non-invasive way. Prior to the development of strategies that make use of NPs, detection options were limited. However, new nanotechnology-based methods allow for the identification of osteoporosis using portable instruments. In particular, research has led to the development of a novel biochip that utilizes gold NPs to identify an osteoporosis-related protein (Singh and Kim, 2012). It has been demonstrated to effectively evaluate the bone condition and precisely detect and recognize the degree of bone injury (Singh and Kim, 2012).

In addition, nanotechnology has been utilized in orthopedics to monitor orthopedic therapies and guide treatment plans (Garimella and Eltorai, 2017b). Some sensors are even enabled to detect the growth of bone or lack thereof and administer extra therapeutic medication as required. (Garimella and Eltorai, 2017b).

Diagnostic instruments enable a physician to identify the origin of an illness and initiate therapy with greater precision and speed. Numerous instruments, devices, materials, and systems, including imaging and sensing techniques, are utilized to aid in the diagnostic process. Examples of diagnostic imaging advancements include Kutsuna and others’ non-invasive soft tissue imaging research, which has shown promise for the future.

Using a nanosecond-pulsed laser and time-resolved laser-induced fluorescence spectroscopy (TR-LIFS), they were efficient in analyzing tissue-engineered cartilage. Utilizing peak fluorescein wavelengths, the researchers were ready to distinguish between two specimens representing variations in collagen replacements and cartilage tension loading (Kutsuna et al., 2010). The identification of bone microfractures is an example of preventative as well as diagnostic imaging. Microcracks are minute fractures before the macroscopic fractures observed in clinical settings.

Bone’s ability to generate microcracks allows it to withstand greater stress before fracture (Murakami et al., 2008). Recent visualization of these microcracks utilizing synchrotron particle accelerator technology has revealed that with and without microcracks, fracture tendencies are distinct. This analysis is diagnostically useful because it reveals the strain properties, for example, intensity, and duration, that led to the fracture (Thurner et al., 2005).

Imaging also assists in distinguishing between conditions. Bone malignancies, such as osteosarcoma and chondrosarcoma, are distinguished using micro- and nano-CT 3D examination (Kuhn et al., 2007; Langheinrich et al., 2008). Nano-CT provides benefits over conventional histological techniques because it is less invasive and maintains dimensions better (Schneider et al., 2007).

Pathogen detection that has colonized a surgical location or intraluminal maneuver is another crucial function of nanotechnology-enabled instruments. Under flow conditions, the Cady laboratory in the Division of Engineering and Nanoscale Science at the University at Albany has demonstrated potential in characterizing bacteriological biofilms.

**FIGURE 5:**
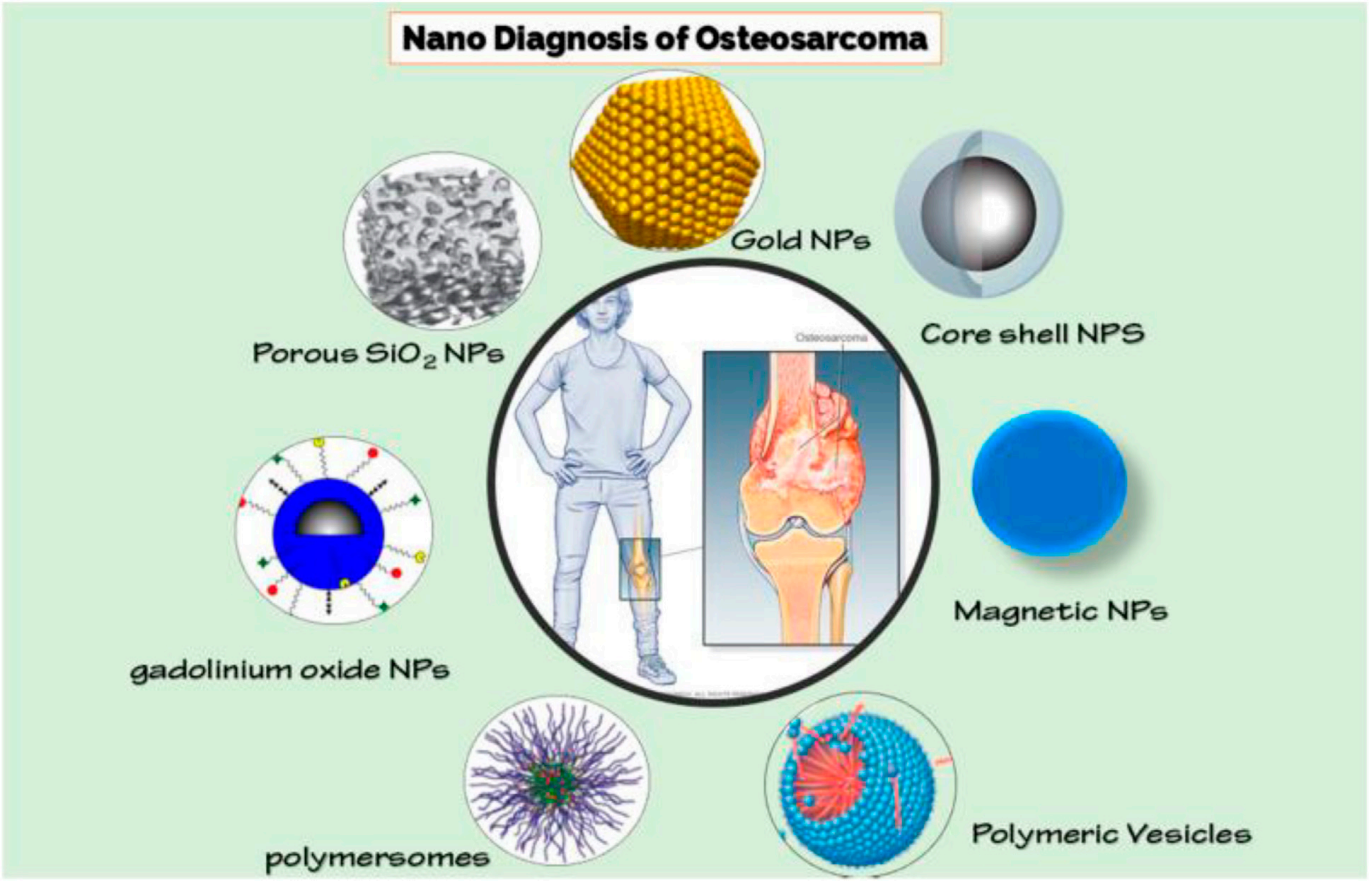
Various Nanoparticles for detecting osteosarcoma (Barani et al., 2021).

To quantify biofilm material properties, atomic force microscopy (AFM) and microfluidic flow platforms were utilized. Dr. Cady has also made advances in disease-detecting systems utilizing disposable DNA sensors, which have a wide variety of potential applications outside of the medical field (Cady et al., 2009; Lui et al., 2009). A previous study (Barani et al., 2021) reviewed and proposed various kinds of NPs for diagnosing osteosarcoma (Figure 5).

### 7.1 Imaging

In the past 10 years, nanotechnology has risen to the forefront of the imaging industry. The orthopedic surgery field, which relies extensively on imaging technology, has a lot to be gained from advancements in the field. Quantum dots are semiconductor particles between 2 and 10 nm in size that emit photons with site-specific imaging capabilities. Quantum dots are useful due to their ability to emit photons during excitation. The photon wavelength released upon excitation is determined solely by the dot size, a parameter that can be precisely adjusted**.** Self-illuminating quantum nanoparticles (NPs) with affixed R. reniformis luciferase (RLuc) (So et al., 2006) are of great relevance to orthopedic operations. Because of endogenous tissue autofluorescence as well as excitation beam diffusion and scattering, conventional imaging with quantum dots excited by external illumination is restricted to superficial depths.

RLuc was used by So et al., to stimulate the quantum dots through bioluminescence resonance energy transfer to circumvent this issue (BRET). BRET permits the nonradiative transfer of chemical energy from a light-emitting enzyme (RLuc) and its substrate to a fluorescent protein. Dots were infused with chemical substrate and RLuc and then injected into a rat model to create a self-contained imaging source. After examination, these quantum dot bioconjugates were shown to be capable of delivering significantly more sensitive deep tissue imaging with quantum dots (Xing et al., 2008; Smith et al., 2009).

Some applications necessitate a greater spatial resolution is required to accurately identify diseased features, whereas the majority of orthopedic imaging applications focus on macroscale differentiation. Osteoporosis, the most prevalent deteriorating condition in the West, is first on the list because it necessitates comprehensive imaging to ascertain both the morphology and density of damaged bone. Currently, the resolving power of conventional computed tomography (CT) is just under 1 mm, which prevents the observation of microscopic features of the bone, including osteocyte lacunae and their connected canaliculi. Quantitative imaging is made possible via a revolutionary technique that utilize three-dimensional density investigations with submicron resolution using psychographic CT. Unlike conventional lenses, pty-chography is founded on refractive microscopy. It gathers micro-diffraction patterns generated when electromagnetic (EM) energy (X-ray in this instance) impacts the specimen using high-speed sensors. In addition to wave density, the data also incorporates the comparative phase of the EM wave. The capacity to quantitatively measure phase variations indicating sample density permits imaging of larger samples while lowering energy deposition compared to conventional high-resolution X-ray techniques (Dierolf et al., 2010).

**FIGURE 6:**
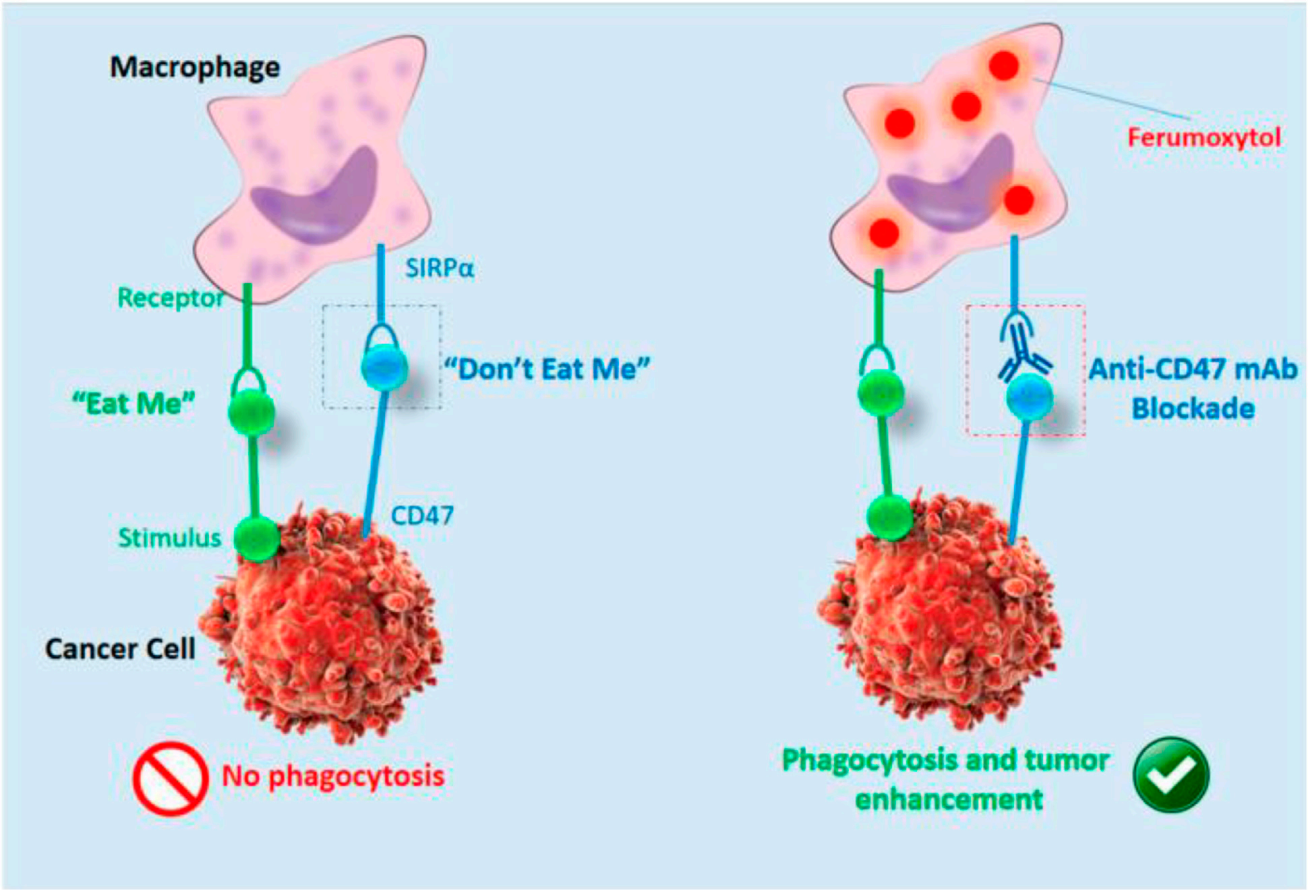
Cartoon showing ferumoxytol-MRI as an imaging approach for CD47 immunotherapy (Mohanty et al., 2019).

Magnetic resonance imaging (MRI) is an additional modality that nanotechnology will enhance. Mohanty et al. (Mohanty et al., 2019) reported that in osteosarcoma, ferumoxytol NPs could enhance MRI and monitor macrophage reaction to CD47 mAb (Figure 6).

Sykova and collaborators are capable of monitoring cellular migration employing T2-weighted MRI images by labeling bone marrow embryonic stem cells (ESCs) and mesenchymal stem cells (MSCs) with super paramagnetic iron oxide (SPIO) NPs (Sykova and Jendelova, 2007). Using this visualization system, they could effectively trace cell migration in models with spinal lesions and cortical. The capability to use tracers to identify the precise locations of neural lesions suggests the probability of future site-specific interventions.

### 7.2 Sensors

Therapeutic interventions and clinical decision-making currently rely heavily on sensing technology. Through nanotechnology, improvements in the accuracy and precision of sensors are rising. The detachment of bone-derived prosthetics stems from one of the leading causes of implant failure in orthopedics. Under physiological conditions, with respect to location and age, the strain on a weight-bearing bone is normally within a reasonable range. Bone tumors, the development of osteoporosis, and prosthesis integration are studied using *in vivo* strain measurements that deviate from normal limits. Metallic foil strain gauges are now available commercially, but their restricted sensitivity, enormous bulk, as well as in absence of long-term biocompatibility pose limitations. Existing strain gauges are being replaced by strain gauges enhanced by nanotechnology in every way (Kumar et al., 2020; Talebian et al., 2020).

For instance, piezoresistive strain sensors on thin films can be integrated into grafts to provide real-time information on the implant’s strength and adherence. Nanocrystalline silicone coatings on composite flexible substrates that are small enough to permit stress with high-sensitivity modeling are used to fabricate these novel strain sensors (Alpuim et al., 2008). A network of these microsensors would enable a real-time, three-dimensional (3D) assessment of force-loading capacities.

Using multi-walled carbon nanotubes (MWCNTs) developed in cavities on a Ti surface is another method for detecting *in situ* bone development. MWCNTs can be used to evaluate the quantity of bone development. MWCNTs measure the tissue’s resistance developing on the grafts; bone hydroxyapatite is conductive, whereas scar tissue and microbes are quite resistant (Liu et al., 2007; Brenner and Ling, 2012). In addition, these MWCNTs increase osteoblast (bone-formation cell) calcium accumulation related to Ti implants presently in use (Sirivisoot and Webster, 2008).

Modern *in vivo* sensors have limitations because they cannot be powered for extended durations. The Lajnef researcher group aims to overcome this restriction by employing piezo strain gauges with ultralow wattage (1 lW) capable of harvesting energy, thereby enabling the graft sensors to operate indefinitely. The prospect of wirelessly transferring this strain and development data to an external receiver through radio-frequency identification (RFID) and shortwave radio (Bluetooth) is also being investigated, so that the data may be employed efficiently in healthcare decision-making (Liu et al., 2007).

## 8 Limitations and future directions

Nanotechnology is relatively new to orthopedic research, diagnostics, and treatment. However, in the short time that it has been studied and implemented, nanotechnology has revolutionized orthopedic treatment science and practice. Nanotechnology offers more precise, better bone growth, and theoretically safer methods of treating the human body, at least regarding infection rates and the need for reoperations. In addition to increasing research on nanotechnology’s current processes, benefits, and hazards, regulatory, manufacturing, and cost barriers should be investigated and addressed. Because of the nature and complexity of nanotechnology products, their production is challenging. The high price of these products can limit their accessibility, and the current regulatory processes can be lengthy, limiting the speed with which research can be implemented. Nanomaterials will be more accessible and their use in orthopedics will be promoted more effectively if these concerns are addressed.

Nanotechnology has the potential to revolutionize the field of orthopedic operation in the coming years. Future directions for research in this area include:

a. Personalized Medicine: Nanotechnology can be used to develop personalized treatments for musculoskeletal disorders. By tailoring treatment to individual patients, outcomes can be improved and complications can be reduced.
b. Regenerative Medicine: Nanotechnology can be used to develop new strategies for tissue regeneration and repair. This could include the use of nanofibrous scaffolds and growth factors to promote the growth of new tissue.
c. Biomimicry: Nanotechnology can be used to create materials that mimic the properties of natural bone tissue. This could lead to the development of more biocompatible implants with improved mechanical properties.
d. Smart Implants: Nanotechnology can be used to develop implants that respond to their environment. This could include the use of sensors to monitor implant performance or the use of drug delivery systems that respond to changes in the body.

## 9 Conclusion

Nanotechnology has the potential to revolutionize the field of orthopedic surgery by improving surgical outcomes, enhancing bone healing, and reducing complications associated with orthopedic procedures. Current applications of nanotechnology in orthopedic surgery include drug delivery, implants, tissue engineering, diagnostics, and infection control. Future directions for research in this area include personalized medicine, regenerative medicine, biomimicry, and smart implants. As the field of nanotechnology continues to evolve, it may play a significant role in diagnosing and treating musculoskeletal disorders.
